# Performance of Swabs, Lavage, and Diluents to Quantify Biomarkers of Female Genital Tract Soluble Mucosal Mediators

**DOI:** 10.1371/journal.pone.0023136

**Published:** 2011-08-12

**Authors:** Charlene S. Dezzutti, Craig W. Hendrix, Jeanne M. Marrazzo, Zhenyu Pan, Lei Wang, Nicolette Louissaint, Sabah Kalyoussef, N. Merna Torres, Florian Hladik, Urvi Parikh, John Mellors, Sharon L. Hillier, Betsy C. Herold

**Affiliations:** 1 Department of Obstetrics, Gynecology, and Reproductive Sciences, University of Pittsburgh, Pittsburgh, Pennsylvania, United States of America; 2 Department of Medicine, University of Pittsburgh, Pittsburgh, Pennsylvania, United States of America; 3 Department of Clinical Pharmacology, Johns Hopkins University, Baltimore, Maryland, United States of America; 4 Department of Medicine, University of Washington, Seattle, Washington, United States of America; 5 Department of Obstetrics and Gynecology, University of Washington, Seattle, Washington, United States of America; 6 Fred Hutchinson Cancer Research Center, SCHARP Statistical Center, Seattle, Washington, United States of America; 7 Department of Pediatrics, Albert Einstein College of Medicine, Bronx, New York, United States of America; University of California Los Angeles, United States of America

## Abstract

**Background:**

Measurement of immune mediators and antimicrobial activity in female genital tract secretions may provide biomarkers predictive of risk for HIV-1 acquisition and surrogate markers of microbicide safety. However, optimal methods for sample collection do not exist. This study compared collection methods.

**Methods:**

Secretions were collected from 48 women (24 with bacterial vaginosis [BV]) using vaginal and endocervical Dacron and flocked swabs. Cervicovaginal lavage (CVL) was collected with 10 mL of Normosol-R (n = 20), saline (n = 14), or water (n = 14). The concentration of gluconate in Normosol-R CVL was determined to estimate the dilution factor. Cytokine and antimicrobial mediators were measured by Luminex or ELISA and corrected for protein content. Endogenous anti-HIV-1 and anti-*E. coli* activity were measured by TZM-bl assay or *E. coli* growth.

**Results:**

Higher concentrations of protein were recovered by CVL, despite a 10-fold dilution of secretions, as compared to swab eluents. After protein correction, endocervical swabs recovered the highest mediator levels regardless of BV status. Endocervical and vaginal flocked swabs recovered significantly higher levels of anti-HIV-1 and anti-*E. coli* activity than Dacron swabs (*P*<0.001). BV had a significant effect on CVL mediator recovery. Normosol-R tended to recover higher levels of most mediators among women with BV, whereas saline or water tended to recover higher levels among women without BV. Saline recovered the highest levels of anti-HIV-1 activity regardless of BV status.

**Conclusions:**

Endocervical swabs and CVL collected with saline provide the best recovery of most mediators and would be the optimal sampling method(s) for clinical trials.

## Introduction

Successful interventions to interrupt the HIV-1 epidemic, particularly among women of reproductive age, are urgently needed. While encouraging advances in the areas of both vaccines and microbicides have recently been reported [1], [2], attempts to explain unsuccessful clinical trials [3], [4], [5], [6], [7] have prompted efforts to better understand how HIV-1 is transmitted during coitus [8] and to develop surrogate biomarkers of microbicide safety [9]. In sub-Saharan Africa, heterosexual sex continues to drive the epidemic, and women are disproportionately infected [10]. The female genital tract (FGT) and in particular the mucosal immune environment may influence HIV-1 acquisition and the effectiveness of HIV-1 prevention modalities.

The FGT is an active immunological site that responds to pathogens (e.g. HIV-1 or other sexually transmitted infections [STIs]) yet accommodates a developing fetus. Sex hormones produce cyclic changes in the FGT that regulate the cellular and innate immune responses [11]. Epithelial cells are sentinel cells and produce a number of soluble mediators that influence immune cell migration and defense against local pathogens including HIV-1 [12]. These mediators are released in response to concurrent/recent STIs and may contribute to the biological and epidemiological synergy between HIV-1 and STIs. The inflammatory response to STIs likely disrupts the protective epithelial barrier and recruits and activates HIV-1 target cells that increase the risk for HIV-1 acquisition [13], [14], [15]. Recruitment of activated target cells plays a role in amplifying the initial HIV-1 transmitted founder virus(es) [16] to a more diverse population fostering local and systemic spread of infection [17]. Endogenous antimicrobial activity, which presumably reflects the collective activity of antimicrobial proteins, cytokines, chemokines, and vaginal microflora, could affect HIV-1 susceptibility [18], [19], [20], [21]. Thus, measurement of individual mediators or functional activity may provide biomarker(s) of risk for HIV-1 acquisition or disease progression as well as serving as surrogate markers of microbicide or mucosal vaccine safety. Most studies have focused on quantifying cytokines, chemokines, and secretory leukocyte protease inhibitor (SLPI) because of the central role of inflammation in promoting HIV-1 shedding [22], [23]. Others have begun to measure endogenous antimicrobial activity and/or concentrations of antimicrobial peptides such as defensins [18], [19]. However, before biomarkers can be identified, tested, and validated, optimal collection method(s) for measuring candidate biomarkers need to be determined [19], [24].

To address this gap, the concentrations of immune mediators (cytokines, chemokines, defensins, SLPI, and lactoferrin [Lf]) and antimicrobial activity (inhibition of *E. coli* and HIV-1) were measured to evaluate whether the site of collection (direct swab of vaginal or endocervical epithelium or cervicovaginal lavage [CVL]), the collection method (Dacron swab, flocked swab or CVL), or diluent of the CVL (saline, Normosol-R, or water) effected recovery of biomarkers of soluble mucosal immunity. Sites and collection methods were chosen based on sampling techniques currently performed in clinical trials. The diluents for the CVL were compared because of specific advantages each may have. Saline is a physiologic solution and commonly obtained in clinics, but the salts may inactivate defensins and interfere with the assessment of the antimicrobial activity [25]. Normosol-R contains gluconate, not present in genital secretions, which may provide a quantitative correction for the dilution of recovered secretions similar to LiCl [26]. Water is widely available, but because it is hypotonic, its use is limited; water can lyse the cells and virus that are targets for testing biological activity. Women with asymptomatic bacterial vaginosis (BV) were included because BV is a common condition associated with an increased risk of HIV-1 acquisition and prevalent among women in sub-Saharan Africa and may impact collection methods [27]. Our interest was to determine the optimal sample collection method(s) for the identification of relevant biomarker(s).

## Methods

### Study population and sample collection

Johns Hopkins University Institutional Review Board approved the study (NA_00036496). Non-pregnant adult women aged 18–45 and without genitourinary symptoms were recruited from the Baltimore, MD metropolitan area. After providing written informed consent, medical history was obtained to exclude women with active menstruation, genitourinary symptoms, or clinically apparent vaginal infections. Previous studies have shown mediator recovery was not affected by the sequence of FGT sample collection [28], [29]. Therefore, among enrolled women, the following were collected in sequence: cervical swab for APTIMA2-Combo assay (GenProbe, San Diego, CA) for *Chlamydia trachomatis* and *Neisseria gonorrhoeae*, Dacron swab (Cardinal Health, McGraw Park, IL) of secretions from lateral vaginal wall for preparation of saline microscopy to evaluate for trichomoniasis and of Gram stain for assessment of Nugent criteria [30]; and for the biomarker analyses, Dacron swab of secretions from lateral vaginal wall, flocked swab (Seacliff Packaging, Inc., Newport Beach, CA) of secretions from lateral vaginal wall, Dacron swab of endocervical secretions, flocked swab of endocervical secretions, and finally CVL with one of three diluents. Women were randomly assigned to receive a 10 ml CVL of saline, tap water, or Normosol-R pH 7.4 (Hospira, Inc., Lake Forest, IL). Normosol-R is a sterile, isotonic balanced salt solution and contains 50.2 mg sodium gluconate per 10 ml. All women with symptomatic BV and STIs detected at screening were excluded and referred for appropriate management. We selected these exclusion criteria because they are typically employed in clinical trials studying prevention of HIV.

### Sample processing

CVL were clarified by centrifugation and supernatants divided into 1 ml aliquots and stored at −80°C. Swabs were placed in 1 ml phosphate-buffered saline (pH 7) and stored at −80°C until processed. Swabs were thawed on wet ice, vortexed for approximately 10 seconds, and compressed against the side of the tube to maximize elution of genital tract secretions. The swab was transferred to a new, sterile 1.8 mL microfuge tube and stored at −80°C. The original tube with the eluent was placed in a refrigerated microfuge centrifuge and clarified at 700×g for 10 minutes. The eluent was divided into 100 µl aliquots and stored at −80°C. Protein concentrations of CVL and swab eluents were determined using the Bradford assay (Sigma-Aldrich, St. Louis, MO).

### Soluble immune mediators

Cytokines (GM-CSF, IL-1β, IL-6, and IL-12p40) and the chemokine IL-8 were quantified in the CVL and swab eluents using Luminex technology (25 µl of sample) (Millipore, Billerica, MA). The sensitivities for these mediators were 9.5 pg/ml, 0.4 pg/ml, 0.3 pg/ml, 0.2 pg/ml, and 10.5 pg/ml, respectively. The mucosal innate immune mediators, Lf (EMD Chemicals, Gibbstown, NJ), SLPI (R&D Systems, Inc., Minneapolis, MN), human β defensins (HβD) 1, 2, and 3 (Alpha Diagnostics, San Antonio, TX), and defensin human neutrophil peptides 1–3 (HNP1–3; HyCult Biotechnology, Uden, The Netherlands) were quantified by ELISA. The limit of detection (LOD) for these commercial assays were >1 ng/ml, <25 pg/ml, 25 pg/ml, 5 pg/ml, 20 pg/ml, and 156 pg/ml, respectively. All ELISAs required 100 µl of sample to perform the assay. Samples often needed to be diluted to be in the linear range of the standard curve. When diluted, reagent diluent for the specific ELISA was used. To standardize levels of the mediators tested, concentrations were corrected by their respective protein concentrations.

### Antimicrobial activity

Antimicrobial activity against *E. coli* or HIV-1 was measured in CVL and swab eluents. For anti-*E. coli* activity, CVL, swab eluent, or control genital tract buffer (GTB; 20 mmol/l potassium phosphate monobasic, 60 mmol/l sodium chloride, 0.2 mg/ml albumin, pH 4.5) was mixed with approximately 6×10^9^ colony-forming units (CFU)/ml of *E. coli* and incubated at 37°C for two hours. The mixtures were then diluted 1000-fold in GTB, mixed with overlay media and plated on agar enriched with trypticase soy broth. Colonies were counted using ImageQuant TL v2005 after an overnight incubation at 37°C. All samples were tested in duplicate and the percent inhibition determined relative to the colonies formed on control plates (800–1000 CFU). For anti-HIV-1 activity, TZM-bl cells were plated at 30,000 cells per well overnight. The cells were treated with CVL, swab eluent, or control buffer and challenged with HIV-1_BaL_ diluted (1∶5) in media. After 48-hour incubation at 37°C, the supernatant was removed, cells were lysed, and luciferase activity was measured in relative light units (Luciferase assay; Promega, Corp., Madison, WI). Mock infected cells served as a negative control. All samples were tested in triplicate in at least two independent experiments.

### Gluconate assay

Gluconate in CVL effluent was assayed using a biochemical two-step enzymatic conversion from gluconate to NADPH with colorimetric detection of product at 340 nm (MegaZyme, Wicklow, Ireland). By comparing the concentration of gluconate in the lavage fluid with the gluconate in the CVL effluent, the dilution of the cervicovaginal fluid was determined.

### Statistical analysis

Descriptive statistics such as median and range were reported for immune mediators and antimicrobial activity. Analysis of variance (ANOVA) was performed to compare concentrations of immune mediators and antimicrobial activity between different diluents of CVL. Linear mixed-effect models that take into account the correlation among repeated measures were used to compare differences in the concentrations recovered by different collection site and method, adjusting for BV (Nugent score ≥7). In this analysis, the three CVL solutions were considered as one type of collection method. To reduce skewness in the distribution, protein-corrected, log-transformed concentrations of all the immune mediators were used in linear mixed model and ANOVA analyses. Spearman rank-order correlation was calculated to assess correlations between mediators. Statistical analyses were performed using SAS 9.2 (SAS, Cary, NC). All statistical tests were two-sided, and *P*≤0.05 indicated statistical significance.

## Results

### Study subjects

**Figure 1:**
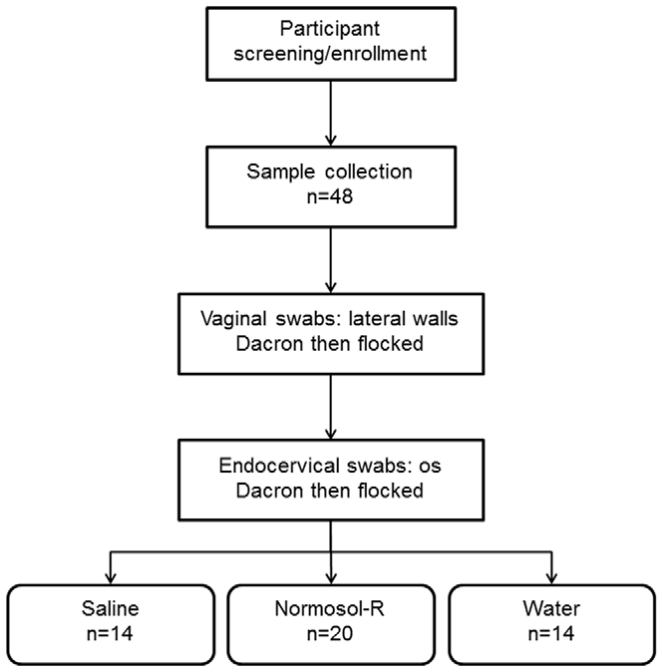
Sample collection algorithm. Sample collection proceeded from the introitus to the cervix. A Dacron swab was rolled 360° along the vaginal lateral wall. A flocked swab was rolled 360° along the opposite vaginal lateral wall. After vaginal swabs, endocervical swabs were taken with first a Dacron swab and then a flocked swab inserted into the cervical os and turned 360°. Finally, the women were randomized to have a CVL collected with 10 ml of saline, Normosol-R, or tap water. Samples were processed as described in the Methods section.

Forty eight women were enrolled. Mean (SD) age was 30.8 (±7.1) years and ranged from 20–45 years. Women indicated the following race: African-American (46%), white (40%), Asian (12%), other (2%). BV was diagnosed in 24/48 (50%) of the women; 23/48 (48%) had no evidence of BV and one was indeterminate based on slide quality. After collection of vaginal and then endocervical swabs, women were assigned to CVL collection with Normosol-R (42%), saline (29%) or tap water (29%) (Figure 1). Overall, recovery of CVL was 8.9±1.8 ml regardless of diluent used.

### Effect of collection method on genital tract secretions and recovery of protein

**Figure 2:**
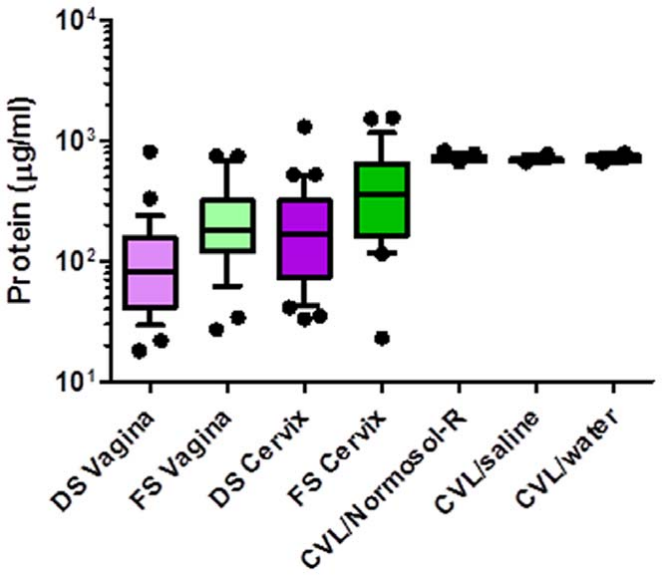
Protein levels in female genital tract secretions collected by swabs and cervicovaginal lavages (CVL). Female genital tract secretions were collected by Dacron swabs (DS) and flocked swabs (FS) from the vagina and the endocervix (cervix) and by CVL using Normosol-R, saline, or water. Protein levels were determined using the Bradford assay. Data are presented as box and whisker plots where the median is the horizontal line through the vertical box which represents the 25–75^th^ percentiles. Values within the 10–90^th^ percentiles are represented by the error bars. Outliers are shown by filled circles. Data were log-transformed and significant changes were determined using linear mixed model and discussed in the results section.

Advantages of CVL collection includes recovery of all material present in the lower genital tract, recovery of a greater volume of material, and overcoming the difficulty of eluting secretions from swabs. Disadvantages include altered mediator concentration due to an unknown dilution factor. To address the disadvantages, the concentration of gluconate in CVL collected in Normosol-R was determined in 20 participants. The recovered cervicovaginal fluid was 235 µl (139.2–304.8 µl; median [range]), a 42-fold dilution of genital tract secretions in a 10 ml CVL. Swabs were not collected in Normosol-R; consequently, the dilution of secretions from a 1 ml swab eluent could not be determined. However, total protein concentration in CVL and swabs was measured. CVL collected significantly higher (*P*<0.001) levels of protein as compared to swabs. The variation in protein content was markedly lower with CVL as compared to swabs (Table 1, Figure 2). The absolute concentrations of each mediator (Table 1 and 2) are presented to provide data on the range of values recovered. To adjust for dilutional variability, the concentration of the soluble mediators was corrected by its protein concentration in the subsequent analyses.

**Table 1:** Levels of immune mediators and endogenous antimicrobial activity in female genital tract samples by collection sites and methods.

Collection method^a^	DS Vag	FS Vag	DS Cer	FS Cer	CVL	p-value^d^
**Protein (µg/ml)**	89^c^ (18–1652)	205 (0–836)	174 (20–4365)	349.5 (18–6804)	694 (664–836)	<0.001
**GM-CSF (pg/ml)**	1.5 (0.5–3.0)	1.6 (0.5–8.1)	1.6 (0.5–11.5)	1.6 (0.5–15.8)	1.6 (0.5–7.1)	<0.001
**IL-1β (pg/ml)**	1.6 (0.1–83.4)	5.1 (0.1–214)	6.1 (0.2–711)	16.1 (0–1140)	5.8 (0.1–482.9)	<0.001
**IL-6 (pg/ml)**	0.3 (0.1–24.0)	0.5 (0.3–140)	17.2 (0.03–564)	42.1 (0.1–578)	8.6 (0.1–92.9)	<0.001
**IL-8 (pg/ml)**	340.0 (11.2–3744)	342.6 (0.1–2883)	1104 (21.3–13557)	1687 (121–5455)	202.3 (2.7–3186.3)	<0.001
**IL-12p40 (pg/ml)**	1.6 (0.1–9.1)	1.6 (1.1–8.4)	2.0 (0.2–17.0)	2.9 (0.2–35.0)	2.8 (0.1–48.5)	<0.001
**Lf (ng/ml)**	254.2 (2.4–2657)	294.2 (6.0–6213)	868.0 (2.0–8472)	1092 (9.8–14467)	282.2 (8.0–4776.7)	<0.001
**SLPI (ng/ml)**	219.5 (3.1–1361)	261.8 (4.7–3304)	411.3 (31.0–3222)	787.1 (68.6–4454)	253.2 (2.8–3908)	<0.001
**HNP1-3 (µg/ml)**	16.2 (4.7–520.5)	16.6 (4.6–507.5)	56.5 (2.4–321.8)	82.8 (5.4–594.8)	48.0 (16.0–4307)	0.02
**Anti-E. coli (% inh.)** b	27.5 (−22–79)	50.5 (4–99)	25 (−11–93)	59.5 (−0.1–100)	78 (20–100)	<0.001
**Anti-HIV-1 (% inh.)**	49 (−188–91)	59 (−42–100)	26.5 (−112-100)	63 (−26–117)	48.5 (−7–99.5)	<0.001

a DS Vag, Dacron vaginal swab; FS Vag, flocked vaginal swab; DS Cer, Dacron endocervical swab; FS Cer, flocked endocervical swab; CVL, cervicovaginal lavage.

b % inhibition – negative values reflect increased growth of *E. coli* or enhancement of infection of HIV-1.

c median (range).

d p-value: Global p-value based on the comparison of protein-adjusted, log-transformed immune mediators and endogenous antimicrobial activities by collection sites and methods.

**Table 2:** Levels of immune mediators and endogenous antimicrobial activity in female genital tract samples by CVL diluents.

Collection method^a^	CVL (Normosol)	CVL (Saline)	CVL (Water)	p-value^e^
Protein (µg/ml)	724.5^c^ (671–836)	688 (668–777)	692 (664–799)	0.43
GM-CSF (pg/ml)	1.5 (0.8–2.4)	1.6 (1.2–7.1)	1.3 (0.5–2.4)	0.02
IL-1β (pg/ml)	5.8 (0.1–483)	8.9 (0.2–204)	3.7 (0.2–39.0)	0.82
IL-6 (pg/ml)	9.5 (0.3–92.9)	9.5 (0.06–33.2)	4.9 (0.3–92.3)	0.85
IL-8 (pg/ml)	223.5 (9.0–3186)	328.3 (30.7–1866)	155.0 (2.7–1470)	0.26
IL-12p40 (pg/ml)	2.8 (0.1–12.2)	2.0 (1.5–48.5)	4.3 (1.6–10.0)	0.70
Lf (ng/ml)	364 (12.4–4777)	309 (50.1–785)	184.2 (8.0–1354)	0.84
SLPI (ng/ml)	284.3 (7.9–2624)	504.9 (13.4–3908)	32.6 (2.8–2710)	0.02
HNP1-3 (µg/ml)	34.8 (18.3–2204)	108 (16.2–1181)	57.1 (16.0–4307)	0.58
HβD-1 (pg/ml)	7732 (1102–16209)	4228 (822–12994)	995 (150–8009)	0.03
HβD-2 (pg/ml)	485 (96–818)	500 (96–633)	161.5 (9.6–538)	0.004
HβD-3 (pg/ml)	710 (182–2962)	1376 (175–11944)	228.5 (121–1113)	<0.001
Anti-*E. coli* (% inh.)^b^	79.5 (20–100)	62 (47–100)	nt^d^	0.52
Anti-HIV-1 (% inh.)	41.3 (−7–63)	68.5 (12–99.5)	nt	0.008

a CVL, cervicovaginal lavage.

b % inhibition – negative values reflect increased growth of *E. coli* or enhancement of infection of HIV-1.

c median (range).

d nt = not tested.

e p-value: Global p-value based on the comparison of protein-adjusted, log-transformed immune mediators and endogenous antimicrobial activity by CVL diluents.

For the CVL collected in 20 women with Normosol-R, the variation in the correction index (individual correction divided by average correction) was smaller for protein (6% coefficient of variation [CV]) when compared to gluconate (23% CV); there was no correlation between protein and gluconate (r = 0.20).

### Recovery of cytokines and the chemokine IL-8

**Figure 3:**
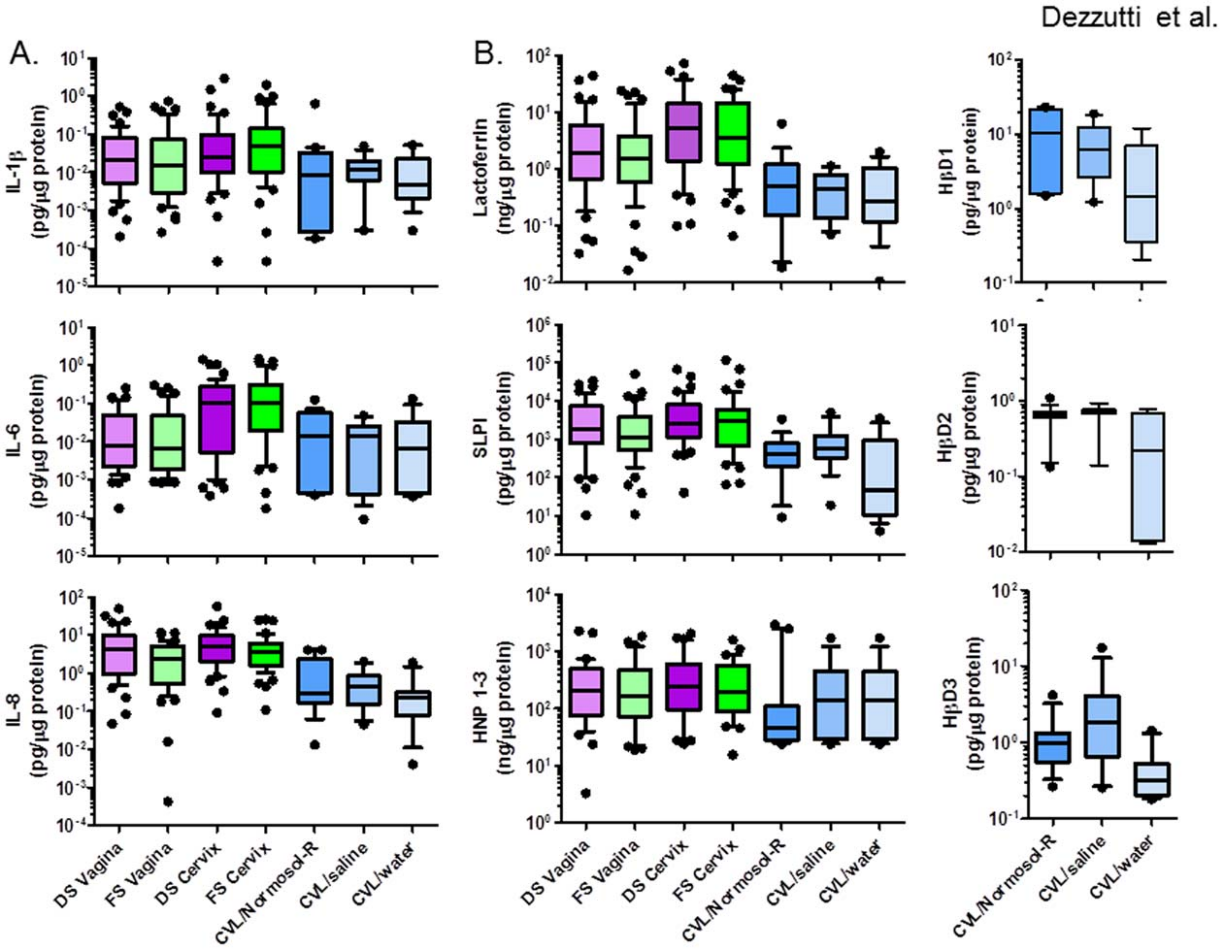
Levels of mediators in female genital tract secretions collected by swabs and cervicovaginal lavages (CVL). Female genital tract secretions were collected by Dacron swabs (DS) and flocked swabs (FS) from the vagina and the endocervix (cervix) and by CVL using Normosol-R, saline, or water. (A) Cytokine levels and IL-8 were measured by luminex technology. (B) Antimicrobial protein levels were measured by commercially available ELISA. β-defensins were measured only in CVL due to limited sample volume from the swabs. Data are presented as box and whisker plots where the median is the horizontal line through the vertical box which represents the 25–75^th^ percentiles. Values within the 10–90^th^ percentiles are represented by the error bars. Outliers are shown by filled circles. Data were log-transformed and significant changes were determined using linear mixed model and discussed in the results section.

Because the levels of GM-CSF and IL-12p40 for the majority of samples were below the LOD regardless of sampling site or specimen collection method, they were not included in the analyses. The recovery of cytokines and IL-8 differed significantly with sampling site and method (*P*<0.001) (Table 1, Figure 3A). Endocervical flocked swabs recovered the highest levels IL-1β and IL-6 and endocervical Dacron swabs recovered the highest level of IL-8. The presence of BV did not affect cytokine or IL-8 recovery from the swab eluents.

Women with BV tended to have better recovery of IL-8 (*P* = 0.02), IL-1β (not significant) and IL-6 (not significant) in CVL collected with Normosol-R as compared to saline and water (Table 2, Figure 2). The diluent type did not significantly impact recovery of mediators in women without BV.

### Recovery of antimicrobial mediators

**Figure 4:**
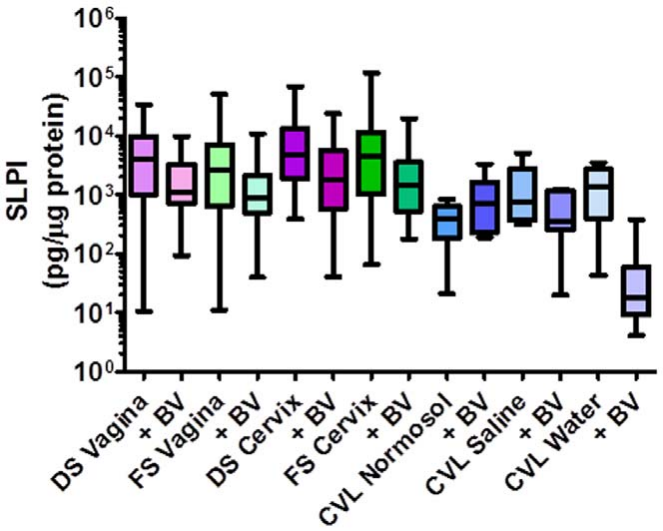
Levels of secretory leukocyte protease inhibitor (SLPI) in female genital tract secretions collected by swabs and cervicovaginal lavages (CVL) in women without or with bacterial vaginosis (BV). Female genital tract secretions were collected by Dacron swabs (DS) and flocked swabs (FS) from the vagina and the endocervix (cervix) and by CVL using Normosol-R, saline, or water. Women with BV (n = 24) had a Nugent score of ≥7. Data are presented as box and whisker plots where the median is the horizontal line through the vertical box which represents the 25–75^th^ percentiles. Values within the 10–90^th^ percentiles are represented by the error bars. Data were log-transformed and significant changes were determined using linear mixed model and discussed in the results section.

Antimicrobial mediator recovery was similar to cytokine and IL-8 recovery. Endocervical swabs recovered the highest levels of Lf (*P*<0.001), SLPI (*P*<0.001), and HNP1–3 (*P* = 0.02) as compared to vaginal swabs and CVL (Table 1, Figure 3B). The presence of BV did not affect Lf or HNP1–3 recovery. However, SLPI levels were significantly reduced in women with BV (*P* = 0.004), but endocervical swabs still recovered higher levels than the other swabs (Figure 4). Due to limited quantities of swab eluents, the levels of β-defensins were not quantified.

The CVL diluents were not significantly different for Lf recovery (Table 2, Figure 2B). CVL collected in Normosol-R and saline recovered significantly higher levels (*P*<0.05) of the three β-defensins as compared to water. BV did not affect the recovery of Lf or the three β-defensins. However, higher levels of SLPI and HNP1–3 were recovered by saline and water, respectively in women without BV, while Normosol-R recovered higher levels of both mediators in women with BV (Figure 4).

### Endogenous antimicrobial activity in FGT secretions

**Figure 5:**
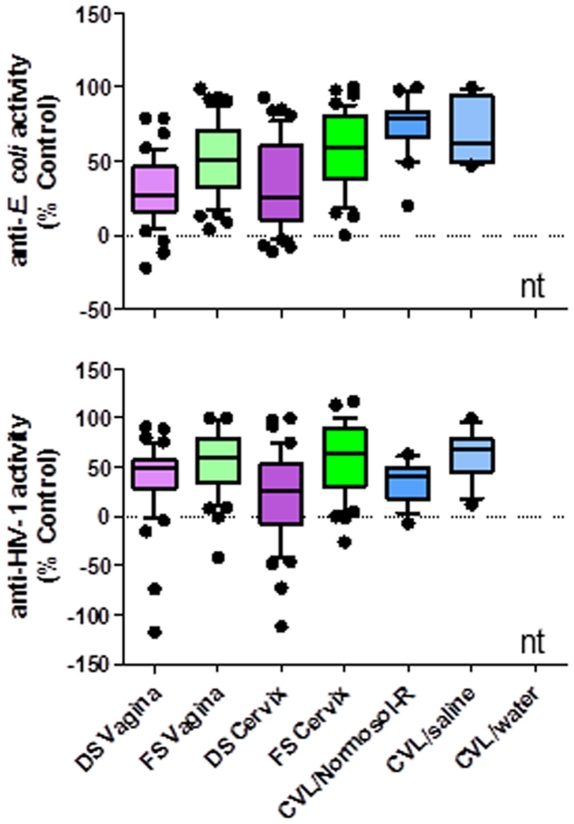
Levels of antimicrobial activity in female genital tract secretions collected by cervicovaginal lavages (CVL) and swabs. Female genital tract secretions were collected by Dacron swabs (DS) and flocked swabs (FS) from the vagina and the endocervix (cervix) and by CVL using Normosol-R, saline, or water. Anti-*E. coli* activity was calculated as the % inhibition of bacterial growth compared to an untreated control. Anti-HIV-1 activity was calculated as the % inhibition of infection as compared to an untreated control. CVL/water was not tested (nt) because water would lyse the pathogens or cells providing non-reportable results. Negative values reflect increased *E. coli* growth or enhanced HIV-1 infection. Data are presented as box and whisker plots where the median is the horizontal line through the vertical box which represents the 25–75^th^ percentiles. Values within the 10–90^th^ percentiles are represented by the error bars. Outliers are shown by filled circles. Significant changes were determined using linear mixed model and discussed in the results section.

The biological activity of the secretions was determined by evaluating the ability of samples to inhibit *E. coli* or HIV-1 in vitro. Because the contributors to inhibition could include mucins and other non-proteinaceous molecules, the anti-microbial activity was not corrected for protein content. CVL sampling with Normosol-R and saline (*P*<0.001) recovered the highest amount of anti-*E. coli* activity compared to the swabs (Table 1 and 2, Figure 5). Flocked swabs (endocervical and vaginal) and CVL collected in saline recovered comparable levels of anti-HIV-1 activity and were higher than the other collection methods (Table 1 and 2, Figure 5). The reasons for significantly higher (*P* = 0.001) recovery of anti-HIV-1 activity among women without BV when CVL was collected in saline as compared to Normosol-R are not known. No differences in HIV-1 infection rates were observed when virus was mixed with Normosol-R or saline prior to infecting TZM-bl cells (data not shown). Water was not tested for antimicrobial activity as the hypotonicity of the sample would lyse the cells and inactivate the virus.

### Correlations between mediators

The antimicrobial activity likely reflects the soluble mediators acting in combination with each other in the FGT, thus correlations between mediators were assessed. IL-1β was modestly correlated with Lf (0.52, *P*<0.0001; r, *P*-value) and HNP1–3 (0.51, *P*<0.0001). IL-6 was modestly correlated with Lf (0.50, *P*<0.0001) and HNP1–3 (0.66, *P*<0.0001). IL-8 was modestly correlated with Lf (0.68, *P*<0.0001), and HNP1–3 (0.67, *P*<0.0001). The anti-*E. coli* activity was weakly, inversely correlated with SLPI (−0.25, *P*<0.0003). None of the mediators correlated to the anti-HIV-1 activity.

## Discussion

Collection of mucosal secretions is commonly performed to test for surrogate markers of product safety and efficacy in studies aimed at testing interventions—such as microbicides and vaccines—to prevent HIV-1. However, biomarkers for these endpoints have proven elusive. Advancement of candidate biomarkers is hampered by the various approaches used to collect genital tract secretions and a paucity of data regarding optimal collection methods, especially about issues that might affect sample collection methods. This study is the first to directly compare the recovery of key candidate biomarkers using different collection methods. Endocervical swabs recovered significantly higher levels of most mediators as compared to vaginal swabs or CVL when controlling for recovered protein.

The finding that higher concentrations of most mediators were recovered from endocervical sampling is consistent with earlier work demonstrating that endocervical cells express higher constitutive levels of cytokines and chemokines compared to vaginal or ectocervical cells [31], [32], suggesting that epithelial cell function may vary at different anatomical sites in the FGT. The stratified squamous epithelium of the lower FGT, which provides a physical barrier to HIV-1, harbors intraepithelial lymphocytes and Langerhans cells. In contrast, the endocervix is lined with a single layer of columnar epithelium covering a lamina propria that is more populated with immune cells than the vaginal lamina propria. This further suggests that endocervical sampling would recover greater amounts of immune mediators secreted by a larger number of immune cells than vaginal sampling.

Interestingly, the swab type (Dacron versus flocked) did not significantly influence recovery of the immune mediators after correcting for protein content. This has important economic implications as flocked swabs are considerably more expensive than Dacron. More protein was recovered from flocked swabs as compared to Dacron swabs, which is consistent with their composition. The flocked swab is made of perpendicular nylon fibers that resemble a soft brush, whereas the Dacron swab is composed of a polyester tip. While the swabs were not weighed, the open weave of the flocked swab likely collected more material. This may explain why flocked swabs were superior to Dacron swabs for detection of STIs [33], [34].

An advantage of collecting mucosal secretions by a swab is the recovery of sample with minimal dilution. However, this may be offset by the difficulty of eluting secretions from the swab and the limited volume of sample recovered, which can restrict the number of assays performed as shown by our inability to test for β-defensins in this study. While logistically more challenging, CVL generates significantly more sample and is commonly performed in clinical trials. Using Normosol-R as a CVL diluent allowed for the measurement of gluconate to quantify the amount of mucosal secretions recovered, which was a median of 235 µl (140–305 µl range) representing an approximate 42-fold dilution in a 10 ml lavage. This finding is consistent with results obtained in other studies using lithium chloride in CVL (300–500 µl) [26], [35] and fluid recovered from the Instead® cup (0.3–0.4 g) [36], [37]. Importantly, the magnitude of the difference among the volume of genital tract secretions recovered between women (slightly >2-fold) was less than the variability in mediators measured by the swabs, suggesting that cervicovaginal fluid volume is not a major source of inter-subject variability in concentrations of mediators or endogenous activity. This is supported by the relatively narrow range of protein concentrations measured in the CVL.

Beyond assessing cervicovaginal fluid volume, lavage diluents containing an internal standard provide absolute concentrations of analytes, not just relative concentration adjustments provided by protein-based correction. The protein correction does not correlate with the gluconate-based correction, suggesting that these are measuring different processes. The cost of an internal standard and its assay must be weighed against the need for improved accuracy and precision in quantitative assessment of cervicovaginal fluid. Due to the consistency of cervicovaginal fluid volume among women, other investigators simply apply a fixed dilutional correction – 42-fold with a 10 mL lavage in our study – to correct for dilution [26], [38], [39].

BV is a condition common among women of reproductive age and is accompanied by a loss of lactobacilli and an increase in anaerobic bacteria. Women with BV have an increased risk of HIV acquisition [27]. The reasons for the increased acquisition are unclear, but BV has been shown to modulate the innate immunity of the FGT [40], [41], [42], [43]. Overall, the ability to recover soluble mucosal immune mediators by swabs was not affected by BV status. Women with BV had similar levels of most mediators with the exception of significantly lower levels of SLPI, which is consistent with others showing BV reduces SLPI levels in the FGT [40], [42]. The reduced SLPI levels were seen in all collection methods with the exception of CVL collected in Normosol-R. Women with BV had generally lower levels of anti-*E. coli* activity as shown by the weak inverse correlation between the two.

While the primary objective of this study was to compare collection methods, the results provide additional data on the range of mediators in the FGT by these diverse methods and the relationship between them (Tables 1 and 2). Concentrations of Lf and HNP1–3 are both produced by neutrophils and correlated with IL-8, which would likely recruit immune cells to the genital tract. No significant correlations between anti-HIV-1 activity and any of the mediators were detected. This is consistent with other studies [18], [44]. Wira and colleagues found comparable anti-HIV-1 activity in CVL from women with and without HIV-1 infection. However, while the anti-HIV-1 activity correlated with the concentrations of HβD2, MIP3α, and anti-HIV gp160 IgG antibodies in HIV-1-infected women, no correlations were found in healthy women [18]. Additionally, we found no correlation between anti-*E. coli* and anti-HIV-1 activity, which is consistent with prior work [45] and suggests that immune mediators differentially contribute to host defense against specific pathogens.

The HIV-1 prevention field has identified several gaps in the ability to define correlates of safety and efficacy in clinical trials [24], [46]. Key to this is the best approach for mucosal sample collection for biomarkers that can be correlated to safety and efficacy outcomes. Findings from this study suggest that endocervical swabs and/or CVL collected in saline may be the optimal approaches. Endocervical sampling recovers the highest levels of most mediators, whereas CVL in saline recovers greater antimicrobial activity. There are limitations to both collection methods. While swabs sample discrete sites in the FGT, the volume of recovered sample and protein is low. Diluting swabs in a lower volume and optimizing methods to elute the secretions from the swabs may help mitigate this limitation. CVL offers a large sample volume to test numerous assays, but the lavage can dilute the mucosal fluid up to 50-fold and low levels of important mediators may be missed. Using both collection methods, if feasible, might provide the best strategy, particularly since the predictive biomarkers have not yet been identified. These results have broad implications for microbicide and mucosal vaccine studies as well as clinical studies designed to identify soluble mucosal mediators associated with HIV-1 risk.

## References

[1] Rerks-Ngarm S, Pitisuttithum P, Nitayaphan S, Kaewkungwal J, Chiu J (2009). Vaccination with ALVAC and AIDSVAX to prevent HIV-1 infection in Thailand.. N Engl J Med.

[2] Abdool Karim Q, Abdool Karim SS, Frohlich JA, Grobler AC, Baxter C (2010). Effectiveness and safety of tenofovir gel, an antiretroviral microbicide, for the prevention of HIV infection in women.. Science.

[3] Pitisuttithum P, Gilbert P, Gurwith M, Heyward W, Martin M (2006). Randomized, double-blind, placebo-controlled efficacy trial of a bivalent recombinant glycoprotein 120 HIV-1 vaccine among injection drug users in Bangkok, Thailand.. J Infect Dis.

[4] Skoler-Karpoff S, Ramjee G, Ahmed K, Altini L, Plagianos MG (2008). Efficacy of Carraguard for prevention of HIV infection in women in South Africa: a randomised, double-blind, placebo-controlled trial.. Lancet.

[5] Buchbinder SP, Mehrotra DV, Duerr A, Fitzgerald DW, Mogg R (2008). Efficacy assessment of a cell-mediated immunity HIV-1 vaccine (the Step Study): a double-blind, randomised, placebo-controlled, test-of-concept trial.. Lancet.

[6] McCormack S, Ramjee G, Kamali A, Rees H, Crook AM (2010). PRO2000 vaginal gel for prevention of HIV-1 infection (Microbicides Development Programme 301): a phase 3, randomised, double-blind, parallel-group trial.. Lancet.

[7] Abdool Karim SS, Richardson BA, Ramjee G, Hoffman IF, Chirenje ZM (2011). Safety and effectiveness of BufferGel and 0.5% PRO2000 gel for the prevention of HIV infection in women.. AIDS.

[8] Hladik F, McElrath MJ (2008). Setting the stage: host invasion by HIV.. Nat Rev Immunol.

[9] Cummins JE, Doncel GF (2009). Biomarkers of cervicovaginal inflammation for the assessment of microbicide safety.. Sex Transm Dis.

[10] UNAIDS/WHO (2010). Report on global AIDS epidemic.. http://www.unaids.org/en/media/unaids/contentassets/documents/unaidspublication/2010/20101123_globalreport_en.pdf.

[11] Wira CR, Fahey JV, Sentman CL, Pioli PA, Shen L (2005). Innate and adaptive immunity in female genital tract: cellular responses and interactions.. Immunol Rev.

[12] Wira CR, Fahey JV, Ghosh M, Patel MV, Hickey DK (2010). Sex hormone regulation of innate immunity in the female reproductive tract: the role of epithelial cells in balancing reproductive potential with protection against sexually transmitted pathogens.. Am J Reprod Immunol.

[13] Corey L, Wald A, Celum CL, Quinn TC (2004). The effects of herpes simplex virus-2 on HIV-1 acquisition and transmission: a review of two overlapping epidemics.. J Acquir Immune Defic Syndr.

[14] McClelland RS, Sangare L, Hassan WM, Lavreys L, Mandaliya K (2007). Infection with Trichomonas vaginalis increases the risk of HIV-1 acquisition.. J Infect Dis.

[15] Smith-McCune KK, Shiboski S, Chirenje MZ, Magure T, Tuveson J (2010). Type-specific cervico-vaginal human papillomavirus infection increases risk of HIV acquisition independent of other sexually transmitted infections.. PLoS ONE.

[16] Keele BF, Giorgi EE, Salazar-Gonzalez JF, Decker JM, Pham KT (2008). Identification and characterization of transmitted and early founder virus envelopes in primary HIV-1 infection.. Proc Natl Acad Sci U S A.

[17] Haaland RE, Hawkins PA, Salazar-Gonzalez J, Johnson A, Tichacek A (2009). Inflammatory genital infections mitigate a severe genetic bottleneck in heterosexual transmission of subtype A and C HIV-1.. PLoS Pathog.

[18] Ghosh M, Fahey JV, Shen Z, Lahey T, Cu-Uvin S (2010). Anti-HIV activity in cervical-vaginal secretions from HIV-positive and -negative women correlate with innate antimicrobial levels and IgG antibodies.. PLoS ONE.

[19] Keller MJ, Guzman E, Hazrati E, Kasowitz A, Cheshenko N (2007). PRO 2000 elicits a decline in genital tract immune mediators without compromising intrinsic antimicrobial activity.. AIDS.

[20] Shust GF, Cho S, Kim M, Madan RP, Guzman EM (2010). Female genital tract secretions inhibit herpes simplex virus infection: correlation with soluble mucosal immune mediators and impact of hormonal contraception.. Am J Reprod Immunol.

[21] Venkataraman N, Cole AL, Svoboda P, Pohl J, Cole AM (2005). Cationic polypeptides are required for anti-HIV-1 activity of human vaginal fluid.. J Immunol.

[22] Cummins JE, Christensen L, Lennox JL, Bush TJ, Wu Z (2006). Mucosal innate immune factors in the female genital tract are associated with vaginal HIV-1 shedding independent of plasma viral load.. AIDS Res Hum Retroviruses.

[23] Mitchell C, Hitti J, Paul K, Agnew K, Cohn SE (2011). Cervicovaginal Shedding of HIV Type 1 Is Related to Genital Tract Inflammation Independent of Changes in Vaginal Microbiota.. AIDS Res Hum Retroviruses.

[24] Jespers V, Harandi AM, Hinkula J, Medaglini D, Le Grand R (2010). Assessment of mucosal immunity to HIV-1.. Expert Rev Vaccines.

[25] Lehrer RI, Ganz T (2002). Defensins of vertebrate animals.. Curr Opin Immunol.

[26] Belec L, Meillet D, Levy M, Georges A, Tevi-Benissan C (1995). Dilution assessment of cervicovaginal secretions obtained by vaginal washing for immunological assays.. Clin Diagn Lab Immunol.

[27] Atashili J, Poole C, Ndumbe PM, Adimora AA, Smith JS (2008). Bacterial vaginosis and HIV acquisition: a meta-analysis of published studies.. AIDS.

[28] Ghanem KG, Johnson RE, Koumans EH, Marrazzo JM, Markowitz LE (2005). Cervical specimen order and performance measures of Chlamydia trachomatis diagnostic testing.. J Clin Microbiol.

[29] Ghanem KG, Koumans EH, Johnson RE, Sawyer MK, Papp JR (2006). Effect of specimen order on Chlamydia trachomatis and Neisseria gonorrhoeae test performance and adequacy of Papanicolaou smear.. J Pediatr Adolesc Gynecol.

[30] Nugent RP, Krohn MA, Hillier SL (1991). Reliability of diagnosing bacterial vaginosis is improved by a standardized method of gram stain interpretation.. J Clin Microbiol.

[31] Fichorova RN, Anderson DJ (1999). Differential expression of immunobiological mediators by immortalized human cervical and vaginal epithelial cells.. Biol Reprod.

[32] Wira CR, Grant-Tschudy KS, Crane-Godreau MA (2005). Epithelial cells in the female reproductive tract: a central role as sentinels of immune protection.. Am J Reprod Immunol.

[33] Chernesky M, Castriciano S, Jang D, Smieja M (2006). Use of flocked swabs and a universal transport medium to enhance molecular detection of Chlamydia trachomatis and Neisseria gonorrhoeae.. J Clin Microbiol.

[34] Krech T, Castriciano S, Jang D, Smieja M, Enders G (2009). Detection of high risk HPV and Chlamydia trachomatis in vaginal and cervical samples collected with flocked nylon and wrapped rayon dual swabs transported in dry tubes.. J Virol Methods.

[35] Mitchell C, Paul K, Agnew K, Gaussman R, Coombs RW (2011). Estimating volume of cervicovaginal secretions in cervicovaginal lavage fluid collected for measurement of genital HIV-1 RNA levels in women.. J Clin Microbiol.

[36] Lai SK, Hida K, Shukair S, Wang YY, Figueiredo A (2009). Human immunodeficiency virus type 1 is trapped by acidic but not by neutralized human cervicovaginal mucus.. J Virol.

[37] Lai SK, O'Hanlon DE, Harrold S, Man ST, Wang YY (2007). Rapid transport of large polymeric nanoparticles in fresh undiluted human mucus.. Proc Natl Acad Sci U S A.

[38] Berneman A, Belec L, Fischetti VA, Bouvet JP (1998). The specificity patterns of human immunoglobulin G antibodies in serum differ from those in autologous secretions.. Infection & Immunity.

[39] Chomont N, Gresenguet G, Hocini H, Becquart P, Matta M (2001). Polymerase chain reaction for Y chromosome to detect semen in cervicovaginal fluid: a prerequisite to assess HIV-specific vaginal immunity and HIV genital shedding.. AIDS.

[40] Draper DL, Landers DV, Krohn MA, Hillier SL, Wiesenfeld HC (2000). Levels of vaginal secretory leukocyte protease inhibitor are decreased in women with lower reproductive tract infections.. American Journal of Obstetrics & Gynecology.

[41] Yudin MH, Landers DV, Meyn L, Hillier SL (2003). Clinical and cervical cytokine response to treatment with oral or vaginal metronidazole for bacterial vaginosis during pregnancy: a randomized trial.. Obstet Gynecol.

[42] Novak RM, Donoval BA, Graham PJ, Boksa LA, Spear G (2007). Cervicovaginal levels of lactoferrin, secretory leukocyte protease inhibitor, and RANTES and the effects of coexisting vaginoses in human immunodeficiency virus (HIV)-seronegative women with a high risk of heterosexual acquisition of HIV infection.. Clin Vaccine Immunol.

[43] Valore EV, Wiley DJ, Ganz T (2006). Reversible deficiency of antimicrobial polypeptides in bacterial vaginosis.. Infect Immun.

[44] Keller MJ, Madan RP, Torres NM, Fazzari MJ, Cho S (2011). A Randomized Trial to Assess Anti-HIV Activity in Female Genital Tract Secretions and Soluble Mucosal Immunity Following Application of 1% Tenofovir Gel.. PLoS One.

[45] Keller MJ, Mesquita PM, Torres NM, Cho S, Shust G (2010). Postcoital bioavailability and antiviral activity of 0.5% PRO 2000 gel: implications for future microbicide clinical trials.. PLoS ONE.

[46] Alliance for Microbicide Development (2006). The Microbicide Development Strategy.

