# Correction: Performance of Swabs, Lavage, and Diluents to Quantify Biomarkers of Female Genital Tract Soluble Mucosal Mediators

**DOI:** 10.1371/annotation/7824df54-632a-48d6-bfbe-42d069d40c6e

**Published:** 2012-01-26

**Authors:** Charlene S. Dezzutti, Craig W. Hendrix, Jeanne M. Marrazzo, Zhenyu Pan, Lei Wang, Nicolette Louissaint, Sabah Kalyoussef, N. Merna Torres, Florian Hladik, Urvi Parikh, John Mellors, Sharon L. Hillier, Betsy C. Herold

Ongoing quality control of the gluconate assay has uncovered problems with assay performance and data processing. Significant, and unpredictable, matrix-interference discovered in an independent re-validation have led the authors to conclude that gluconate is not a reliable analyte for measuring cervicovaginal fluid dilution. As a result, gluconate adjusted cervicovaginal fluid volumes reported in the manuscript are incorrect and are not correctible. Other findings and conclusions of the study remain unaffected. 

